# Association between body mass index and muscularity in healthy older Japanese women and men

**DOI:** 10.1186/1880-6805-32-4

**Published:** 2013-03-04

**Authors:** Hiroaki Kanehisa, Tetsuo Fukunaga

**Affiliations:** 1 National Institute of Fitness and Sports in Kanoya, 1 Shiromizu, 891-2393, Kanoya, Kagoshima, Japan

**Keywords:** Muscle thickness, Estimated muscle mass, B-mode ultrasonography

## Abstract

**Background:**

Body mass index (BMI), expressed as the ratio of body mass to height squared (kg/m^2^), involves not only fat but also lean mass. The present study aimed to clarify how BMI is associated with total muscle mass (TMM) in older Japanese women and men.

**Findings:**

Using a B-mode ultrasound apparatus, muscle thickness was measured at nine sites (forearm, upper arm anterior and posterior, thigh anterior and posterior, lower leg anterior and posterior, abdomen, and subscapular) for 346 women (BMI 16.40 to 33.11 kg/m^2^) and 286 men (BMI 16.86 to 31.18 kg/m^2^) aged 60.0 to 79.5 yrs. TMM was estimated using the product of the sum of the muscle thicknesses at the nine sites with height as an independent variable. For both sexes, the estimated TMM relative to height squared was significantly correlated with BMI (*r* = 0.688, *P<*0.0001 for women; *r* = 0.696, *P<*0.0001 for men), but the percentage of the estimated TMM in body mass was not.

**Conclusion:**

These results indicate that, for older Japanese women and men, BMI is a simple and convenient index for assessing total muscularity.

## Background

Body mass index (BMI) is widely used for assessing overweight and obesity [1]. However, the association between BMI and the percentage of body fat mass (%BF) in body mass is influenced by age, sex, and ethnicity [2-7]. Some studies have suggested that the diagnostic accuracy of BMI to detect excess body adiposity diminishes with increasing age of the person being assessed [8,9]. Romero-Corral *et al*. [9] found that the correlation between BMI and %BF was lower in older than in younger subjects, but the correlation between BMI and lean mass was similar across age groups. Micozzi and Harris [10] suggested that, based on anthropometric measurements, the ratios of body mass to height^2^ and height^1.5^ in men and women, respectively, are more closely correlated with estimates of body fat in younger than in older adults, and with estimates of muscle mass in older than in younger adults. This suggests that, for the elderly individual, BMI may be a simple and convenient index for assessing muscularity. This assumption has already been examined using populations aged 60 and over [10-13]. Iannuzzi-Sucich *et al*. [11] indicated that BMI is a strong predictor of skeletal-muscle mass in older Caucasian women and men. In their study, using dual X-ray absorptiometry (DXA), BMI was shown to account for 48% and 50% of the variance in appendicular skeletal muscle mass in women and men, respectively. However, in other studies, the *r*^2^ values between BMI and muscle mass were not so high (*r*^2^ = 0.22 to 0.40) [10,12,13]. The reasons for these lower *r*^2^ values are unknown, but it might be due to the fact that the previous studies used muscle girth or area estimated from the anthropometric data of the upper arm [10,13] or the upper arm and calf [12].

Gallagher *et al*. [4] reported that the middle-aged and older Japanese population had a higher %BF for any given BMI than did white and African-American populations. Considering this, whether the findings of Iannuzzi-Sucich *et al*. [11] can be applied to the elderly Japanese population remains unclear. Furthermore, it seems strange that the previous studies cited above correlated the measures obtained from a limited number limbs with BMI, which is an index representing the total body. To our knowledge, no study has examined how total muscle mass is associated with BMI in elderly individuals. Thus, the present study aimed to examine the association between BMI and muscle mass in elderly Japanese individuals of both sexes. To this end, we determined muscle thicknesses at nine sites of the body using B-mode ultrasonography. The product of the sum of muscle thicknesses at these nine sites with height was shown to have a high correlation with total muscle mass (TMM) in a sample of Japanese women and men [14]. The equation with the product of the two variables as an independent variable for predicting TMM, developed in the previous study [14], has been successfully used to examine age-related muscle loss in Japanese men and women aged 20 to 95 years [15]. Using of the prediction equation, therefore, the present study estimated TMM for an older population. We hypothesized that, for the elderly population, the estimated TMM relative to height squared would be significantly correlated with BMI, but its value relative to body mass would not.

## Methods

### Ethics approval

This study was approved by the Ethics Committee of the Graduate School of Arts and Sciences, University of Tokyo, Japan, and was consistent with the institutional ethics requirements for human experimentation in accordance with the Declaration of Helsinki. The subjects were fully informed of the purpose and risks of the experiment, and gave their written informed consent.

### Subjects

A group of 346 women and 286 men aged 60.0 to 79.5 years voluntarily participated in this study. None of the subjects was or had been an athlete. Moreover, none was using walking sticks or other walking aids ,and all were functionally independent in daily life. In addition, no participant was on an extreme diet or using any major medications, such as chemotherapy, cardiac, respiratory, or antipsychotic drugs. The mean ± standard deviation (SD) for age, height, body mass, and BMI are presented in Table 1.

**Table 1:** **Descriptive data on the measured variables**^a,b^

**Variables**	**Women, n = 346**	**Men, n = 286**
Age, years	69.4 ± 4.9	69.8 ± 4.7
Height, cm	150.1 ± 5.4	163.4 ± 6.0
Body mass, kg	53.6 ± 7.1	63.5 ± 8.
BMI, kg/m^2^	23.77 ± 2.77	23.76 ± 2.58
Muscle thickness, mm		
Forearm	19.1 ± 3.4	22.6 ± 4.2
Upper arm anterior	28.4 ± 3.8	33.3 ± 4.6
Upper arm posterior	26.9 ± 4.7	32.8 ± 5.1
Thigh anterior	38.1 ± 5.6	42.1 ± 6.1
Thigh posterior	56.6 ± 7.0	61.9 ± 7.5
Lower leg anterior	25.9 ± 2.8	28.7 ± 3.2
Lower leg posterior	59.5 ± 4.6	66.0 ± 5.6
Subscapular	18.2 ± 4.3	21.1 ± 4.7
Abdomen	7.6 ± 1.7	10.4 ± 2.1
SMT^c^, mm	280.3 ± 21.4	319.8 ± 27.7
TMM^d^, kg	13.8 ± 2.6	20.8 ± 3.7
TMM/ht^2^,^e^ kg/m^2^	6.10 ± 0.99	7.77 ± 1.15
%TMM^f^	25.7 ± 3.1	32.7 ± 3.5

^a^Values are mean ± SD.

^b^All listed variables except for age and BMI were significantly greater (*P*<0.0001) in men than in women.

^c^Sum of the muscle thickness values at nine sites.

^d^Estimated total muscle mass.

^e^TMM relative to height squared.

^f^Percentage of TMM in body mass.

### Muscle thickness measurements

Muscle thickness was measured at nine sites (forearm, upper arm anterior and posterior, thigh anterior and posterior, lower leg anterior and posterior, abdomen, and subscapular) on the right side of the body, using a real time B-mode ultrasound apparatus (SSD-500, Aloka Co., Tokyo, Japan). The position of the subjects during the ultrasonographic measurements, the site selected for obtaining cross-sectional images, and determination of muscle thickness at each site were the same as those described in a previous study [15]. The sum of muscle thicknesses at the nine sites (SMT) was used to estimate TMM. TMM was estimated using the prediction equation developed by Sanada *et al*. [14]: TMM (kg) = 0.687 × SMT (cm) × body height (m) − 15.122 (*R*^2^ = 0.96, SEE = 1.1 kg). In addition to the absolute value, the estimated TMM relative to height squared (TMM/ht^2^, kg/m^2^) and the percentage of the estimated TMM in body mass (%TMM) were calculated, and used to examine how muscularity and the relative distribution of muscle mass within body mass are associated with BMI.

### Statistics

Descriptive values are presented as mean ± SD. An unpaired Student’s *t*-test was used to test the differences between men and women in the measured variables. A simple linear regression analysis was used to calculate the coefficient of correlation between BMI and TMM/ht^2^ or %TMM. The probability level for statistical significance was set at *P*<0.05.

## Results

**Figure 1:**
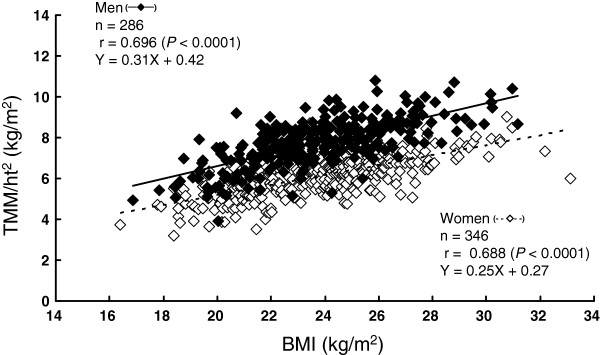
**Relationship between BMI and TMM/ht**^**2**^**.**

**Figure 2:**
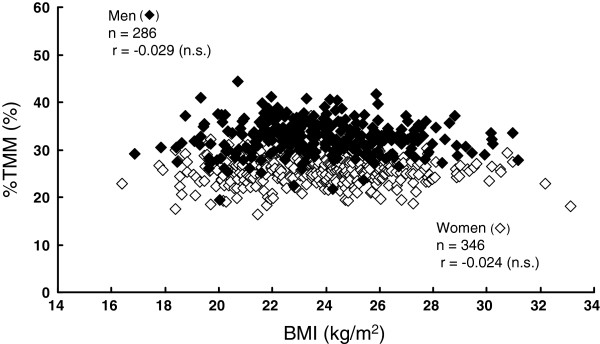
Relationship between BMI and %TMM.

All measured variables except for age and BMI were significantly greater in men than in women (Table 1). TMM/ht^2^ was significantly correlated with BMI in both women (*r* = 0.688, *P<*0.0001) and men (*r* = 0.696, *P<*0.0001) (Figure 1). However, %TMM was not significantly associated with BMI in either sex: *r* = −0.024 (*P*>0.05) for women and *r* = −0.029 (*P*>0.05) for men (Figure 2).

## Discussion

The current results support our hypothesis regarding the association between BMI and muscle mass, and indicate that, for the elderly person, BMI is an index that assesses total muscularity rather than the relative distribution of muscle mass within the total body. Based on the *r*^2^ values, BMI accounted for 47% and 48% of the variance in TMM/ht^2^ in women and men, respectively. These values are higher than those reported in previous studies using anthropometric approaches for predicting muscle size [10,12,13], and are comparable those in the study of Iannuzzi-Sucich *et al*. [11] who used DXA to determine appendicular skeletal muscle mass. In the present study, we estimated the total muscle mass by using a prediction equation developed by Sanada *et al*. [14]. Based on the report of Sanada *et al*. [14], the accuracy of the ultrasonography prediction model for estimating TMM is greater than that of anthropometric and bioelectrical impedance prediction models, and is similar to that of DXA prediction models and whole-body 40K counting. This may explain the aforementioned difference in the observed *r*^2^ between the current study and previous reports using anthropometric model. However, it should be noted that the number of subjects in our study categorized as underweight (BMI*<*18.50 kg/m^2^) or obese (30.00 kg/m^2^ or higher) [1] was small (23; 3.6%). The correlation coefficient of the associations between BMI and TMM/ht^2^ was found to be lower for subjects with BMI greater than 25 (women: *r* = 0.468, *P<*0.001, men: *r* = 0.448, *P<*0.0001) than for subjects with BMI less than 25 (women: *r* = 0.660, *P<*0.0001, men: *r* = 0.630, *P<*0.0001). This implies a limitation on the use of BMI as an index for assessing muscularity in elderly individuals with relation to the magnitude of BMI. Further studies examining individuals categorized as either underweight or obese are needed to clarify this finding.

## Abbreviations

%BF: Percentage of body fat mass in body mass;BMI: Body mass index;TMM: Total muscle mass;SMT: Sum of muscle thicknesses at the nine sites;TMM/ht2: Estimated TMM relative to height squared;%TMM: Percentage of the estimated TMM in body mass

## Competing interests

The authors declare that they have no competing interests.

## Authors’ contributions

HK participated in study design, helped acquire funding from the Ministry of Education, Culture, Sports, Science and Technology, helped coordinate research activities, performed statistical analysis, and drafted the manuscript. TF participated in study design and coordination, and drafted the manuscript. Both authors read and approved the final manuscript.
